# Shared decision making in sarcopenia treatment

**DOI:** 10.3389/fpubh.2023.1296112

**Published:** 2023-11-22

**Authors:** Kang An, Zengxiang Wu, Yu Qiu, Mengjia Pan, Lin Zhang, Zhenmei An, Shuangqing Li

**Affiliations:** ^1^General Practice Ward/International Medical Center Ward, General Practice Medical Center, National Clinical Research Center for Geriatrics, West China Hospital, Sichuan University, Chengdu, Sichuan, China; ^2^Department of Endocrinology and Metabolism, West China Hospital, Sichuan University, Chengdu, Sichuan, China

**Keywords:** shared decision making, patient-centered care, partnership of care, patient values and preferences, older adults, sarcopenia

## Abstract

The implementation of shared decision making (SDM) in management of sarcopenia is still in its nascent stage, especially compared to other areas of medical research. Accumulating evidence has highlighted the importance of SDM in older adults care. The current study overviews general SDM practices and explores the potential advantages and dilemmas of incorporating these concepts into sarcopenia management. We present common patient decision aids available for sarcopenia management and propose future research directions. SDM can be effectively integrated into daily practice with the aid of structured techniques, such as the “seek, help, assess, reach, evaluate” approach, “making good decisions in collaboration” questions, “benefits, risks, alternatives, doing nothing” tool, or “multifocal approach to sharing in shared decision making.” Such techniques fully consider patient values and preferences, thereby enhancing adherence to and satisfaction with the intervention measures. Additionally, we review the barriers to and potential solutions to SDM implementation. Further studies are required to investigate measurement and outcomes, coordination and cooperation, and digital technology, such as remote SDM. The study concludes that sarcopenia management must go beyond the single dimension of “Paternalism” choice. Integrating SDM into clinical practice offers promising opportunities to improve patient care, with patient-centered care and partnership of care approaches positively impacting treatment outcomes.

## Background

Medical decision making has undergone significant changes in recent decades, leading to a major paradigm shift in geriatric medicine. The traditional patient-physician relationship under a paternalistic care model has evolved into a more collaborative and participatory interaction between patients and healthcare professionals. This transformation has led to the emergence of shared decision making (SDM) as a key approach that emphasizes active patient involvement and considers their values and preferences to foster a comprehensive understanding of treatment options and potential risks (1–3). SDM partnerships gradually extend into large networks involving patients, family members, healthcare teams, and nonprofessional community organizations.

Healthy aging is an integral component of advancing the “Healthy China” strategy (4). In 2015, China introduced the concept of establishing a hierarchical medical treatment system with the objective of managing “serious illnesses in hospitals, minor illnesses in the community, and rehabilitation into the community” (5). Diagnosed patients represent only “the tip of the iceberg,” among a large number of undiagnosed patients within the community. An epidemiological investigation of sarcopenia in the Chinese population revealed a prevalence rate ranging from 9.9 to 40.4% among individuals aged 60 years and older (6–8). The prevalence rate of possible sarcopenia in community-dwelling older Chinese adults is 46.0% (9). The primary treatments for sarcopenia include rehabilitation exercises and nutritional support (8). Although sarcopenia guidelines emphasize the importance of geriatric medicine experts, a multidisciplinary approach is required to select appropriate intervention methods based on patients’ experiences, values, and expectations. While SDM has been rapidly incorporated into chronic disease fields such as diabetes and hypertension, specific adaptations for geriatric syndrome are relatively lagging in clinical settings.

Several international guidelines, such as EWGSOP2 by European Working Group on Sarcopenia in Older People (10), ICFSR 2018 by International Clinical Practice Guidelines for Sarcopenia (11), and AWGS 2019 by Asian Working Group for Sarcopenia (12), do not offer specific guidance for integrating SDM into routine clinical practice. This research provides a brief overview of current SDM practices; discusses the scenarios, considerations, and tips of its implementation for sarcopenia management; explores helpful SDM tools; and identifies strategic priorities, and future challenges—what do we need to integrate SDM in the daily practice of sarcopenia management.

## Role of SDM in sarcopenia management

Community is the most important site for improving the overall healthcare of the older adults (13), particularly for those with sarcopenia or probable sarcopenia. Primary care plays a crucial role in age-related conditions and provides coordinated, comprehensive, continuous, and accessible care (14). An effective primary care model requires a patient-centered approach, effective communication, and cultural competence in evidence-supported decision making, and collaborative care (15).

The management of sarcopenia requires interdisciplinary collaboration. However, the predominant approach is the “geriatricians-driven” model (16). Challenges arise by the integration of healthcare teams with diverse medical backgrounds, including geriatricians, general practitioners, rehabilitation therapists, and nurses. The complexity of the factors contributing to sarcopenia and the diverse presentations of each individual’s condition emphasizes the crucial role of a multidisciplinary approach to SDM in effective medical management. SDM should be initiated during the early stages of risk factor identification and should continue throughout the course of high-quality care (17). Implementing strategies such as preemptive patient education, standardized and regular assessments, adapting treatments based on patient preferences, and fostering interdisciplinary collaboration can deliver optimal individualized care (18). For individuals with specific sarcopenia conditions, such as cardiopulmonary impairment, obesity, and balance disorders, tailored progressive resistance training programs should be implemented (8).

Combined exercise and nutritional intervention is an effective approach for enhancing muscle mass, strength, and physical performance in individuals with sarcopenia (19). Studies on sarcopenia in older Chinese adults have revealed that a 12-week intensified lifestyle intervention incorporating nutritional supplementation and resistance training program significantly improves muscle mass (20, 21). The Chinese expert consensus on the prevention and intervention for older adults with sarcopenia (2023), similar to international guidelines, advocates combining nutritional intervention with exercise training programs, including resistance training, aerobic exercises, and balance training with resistance training as the primary treatment (8). Given the potential coexistence of malnutrition and sarcopenia, older adults patients with sarcopenia should undergo nutritional assessments using appropriate evaluation scales (22). Appropriate Nutritional supplementation should be provided to such patients (22). Proteins and amino acids are among the most promising dietary supplements (23, 24). Tai Chi improves balance control (8). The Chinese consensus recommends 24-form simplified Tai Chi as the preferred traditional exercise (8). However, insufficient evidence supports the use of drugs and traditional Chinese medicine for the treatment of sarcopenia (8, 25).

## SDM for sarcopenia intervention providers

Situations in which SDM for sarcopenia management may be applicable can be categorized into four scenarios: simple situations, such as discussing the patient’s condition; complex situations involving special populations with specific needs; discrete situations that include considering various intervention options; and situations that require continuous care management, such as formulating long-term treatment and care plans. Overall, the process of SDM revolves around dyadic patient-doctor interactions whereby the doctor (i) presents the merits and demerits of all treatment options, (ii) elicits patient values and preferences, (iii) provides opportunities for the patient to clarifying any queries, and (iv) makes a recommendation that is respectful of the clinical context and individual patient preferences (1). Importantly, the SDM is an iterative process. As initial goals are met or as patients’ status or treatment goals change, the process may start anew (1, 18). When presenting patients with treatment recommendations, the discussion should be of the access type in an unbiased manner and should consider the advantages and disadvantages of each type.

First, patient knowledge and awareness of sarcopenia are crucial for establishing appropriate expectations and treatment preferences. The multifocal approach to sharing in shared decision making (MAPPIN’SDM) is an integrated SDM measurement instrument that considers patient, physician, and observer perspectives, assessing both behavior and perception related to SDM (26). MAPPIN’SDM has been successfully used to identify patients’ needs and expectations in rehabilitation settings, thereby facilitating early expectation management and patient education (27). Expectations may increase patient satisfaction. MAPPIN’SDM is also a valuable tool for assessing and comparing patient engagement and the quality of decision-making communication throughout interventions, contributing to the improvement of SDM practices among healthcare teams (26).

**Figure 1:**
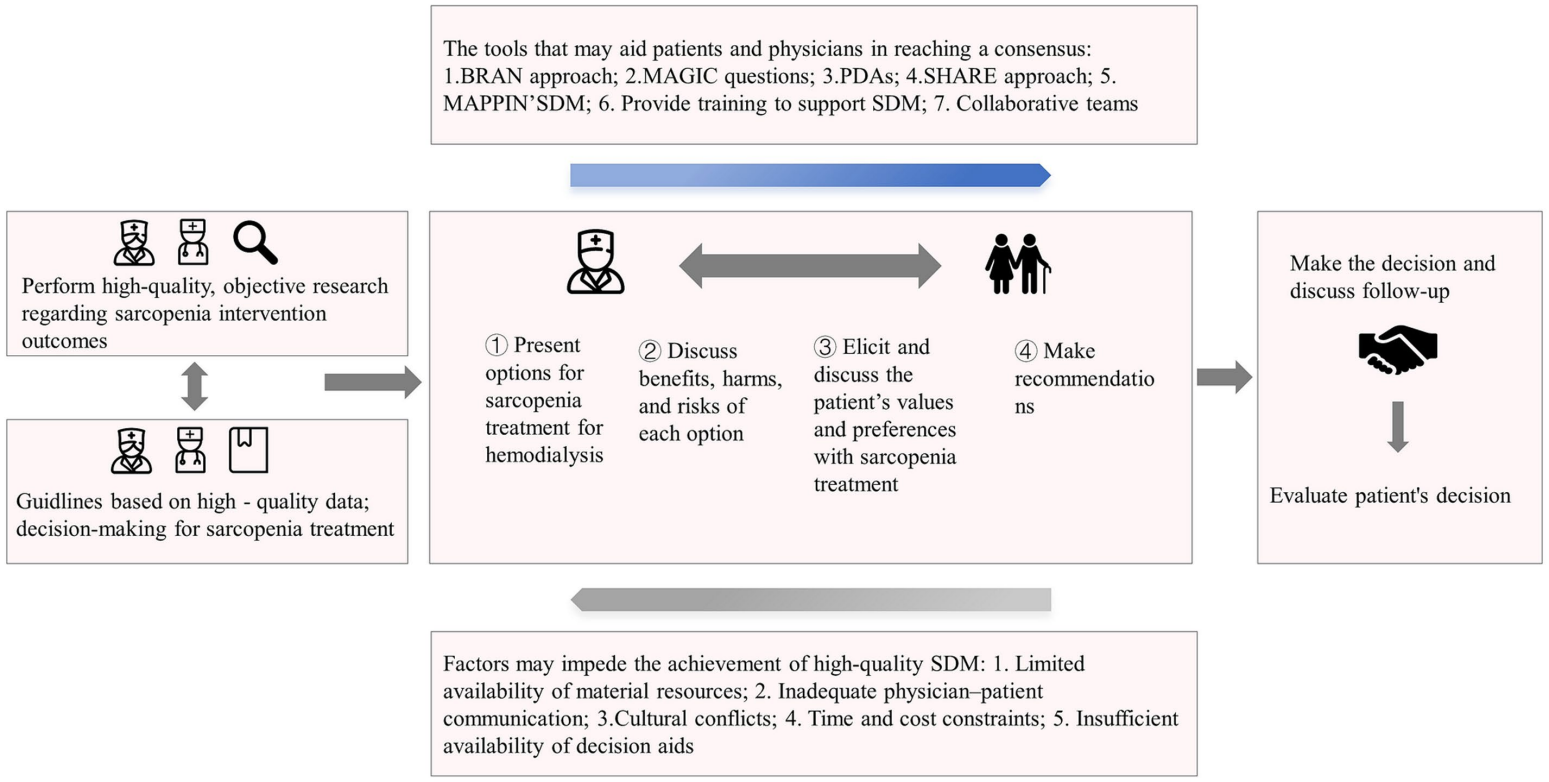
Fundamentals and influencing factors associated with shared decision making for sarcopenia treatment.

Healthcare providers can adapt their communication approaches to engage in comprehensive discussions with patients regarding sarcopenia, including its definition, potential risks, treatment options, benefits, and expected outcomes. While providing treatment recommendations, healthcare teams should seek to understand patients’ values and preferences regarding treatment options. The “seek, help, assess, reach, evaluate” (SHARE) approach can guide the early decision-making pathway in sarcopenia intervention (28). Patients can also use accessible patient-driven decision-making tools, such as the “making good decisions in collaboration” (MAGIC) questions (29) or the “benefits, risks, alternatives, and doing nothing” (BRAN) tool (30), to make informed and considerate decisions about their own care (Table 1). Through a collaborative process involving healthcare teams, patients, and their families, a mutual decision is made that is subject to periodic reviews based on clinical changes. We can use a decision pathway from a unidirectional physician-to-patient practice to a bidirectional collaborative health team-patient practice (Figure 1). Tools that facilitate or hinder consensus between patients and physicians are depicted in the upper and lower boxes within the patient-doctor dyad. Addressing these factors is crucial for creating suitable conditions for promoting effective SDM.

**Table 1:** Recommended tools for daily clinical practice of shared decision making: SHARE (28), MAGIC (29), and BRAN (30).

Tool	Essential steps	Sample discussion points and questions for the example of formulating a treatment plan for older adults patients with sarcopenia
SHARE (28)	1. Seek patients’ participation	“There are many options that I would like to explore and discuss with you.”
	2. Offer help during the acquisition of knowledge regarding treatment options	““It is your decision, and I’m here to help you understand the various options available for sarcopenia, including aerobic exercise, balance training, resistance training, appropriate nutritional supplementation, traditional Chinese sports.”
	3. Assess values and preferences among patients	“What level of improvement are you expecting? How much control do you want to have over your treatment? Are there specific aspects of the treatment you are concerned about, such as efficacy, risks, costs, treatment duration, or complexity?”
	4. Reach a participatory decision	“After evaluating the advantages and disadvantages, and taking your values into account, we have selected two approaches. These include suitable nutritional supplementation and traditional Chinese sports. How do you perceive this plan? We can delve into this decision more deeply and make any necessary adjustments.”
	5. Evaluate the decision	“Can we talk next [appropriate timeframe] to see how you are doing? If you do not feel things are improving, please schedule a follow-up visit so we can plan a different approach. If you encounter any challenges or barriers in adhering to the prescribed interventions, we will help you overcome these obstacles effectively.”
MAGIC (29)	1. What are the available options?	“I have received a diagnosis of sarcopenia. What intervention options are available for me to consider?”
	2. What are the possible benefits and risks of those options?	“What are the potential benefits and risks associated with each intervention? Are there any exercises that I can perform independently without needing to visit the hospital? Can these interventions lead to a rapid increase in muscle mass?”
	3. How likely are the possible benefits and risks of each option to occur?	““Given my comorbid conditions of hypertension and coronary heart disease, does resistance training pose any risks for me? How likely am I to benefit from Tai Chi?”
BRAN (30)	1. Benefits	1. What advantages can I derive from balance training?
	2. Risks	2. What potential risks are associated with balance training?
	3. Alternatives	3. Are there alternative treatments available? What are the potential benefits and risks of these options, including alternatives like traditional Chinese medicine?
	4. Consequences of no treatment	4. What could potentially occur if I opt not to pursue treatment? How will it affect my daily life, and what are the risks, such as an increased risk of falls?

Sarcopenia, a comorbid disease with high multimorbidity and severity, is highly prevalent in patients with cardiovascular diseases, chronic obstructive pulmonary disease, osteoporosis, chronic kidney disease, and cancer (18, 31–33). Healthcare teams should be extremely cautious when evaluating patients with comorbidities. However, several points require further consideration. First, a comprehensive assessment of the patient’s medical history and current health status is essential for identifying and understanding all existing comorbidities. The first step in treating sarcopenia is to determine and treat the underlying cause (34). For example, exercise and managing calorie intake may contribute to the reduction of myosteatosis in obese patients (35). Second, physicians should be attentive to the potential interactions between sarcopenia treatment and the management of other health issues. Currently, whether patients with osteoporosis are suitable for resistance exercises (36). Third, healthcare professionals should consider the patients’ overall health goals and preferences when formulating treatment plans to address both sarcopenia and comorbidities.

Such instances may arise when patients are hesitant to undergo interventions. During SDM, the healthcare team should use strategies to increase patient involvement in health behavior changes, including the five As, five Rs, and motivational interviewing, while actively providing relevant information and recommendations (37). The team can engage in collaborative discussions with patients regarding the potential implications and consequences of sarcopenia to enhance their understanding of its severity and the importance of treatment. The team should consistently uphold patient autonomy and decision-making authority and address any misconceptions. If patients refuse treatment, the team and patients can work together to establish a monitoring plan, regularly assess muscle condition, and provide the necessary education and support.

## Remaining challenges in establishing an SDM culture in sarcopenia

**Figure 2:**
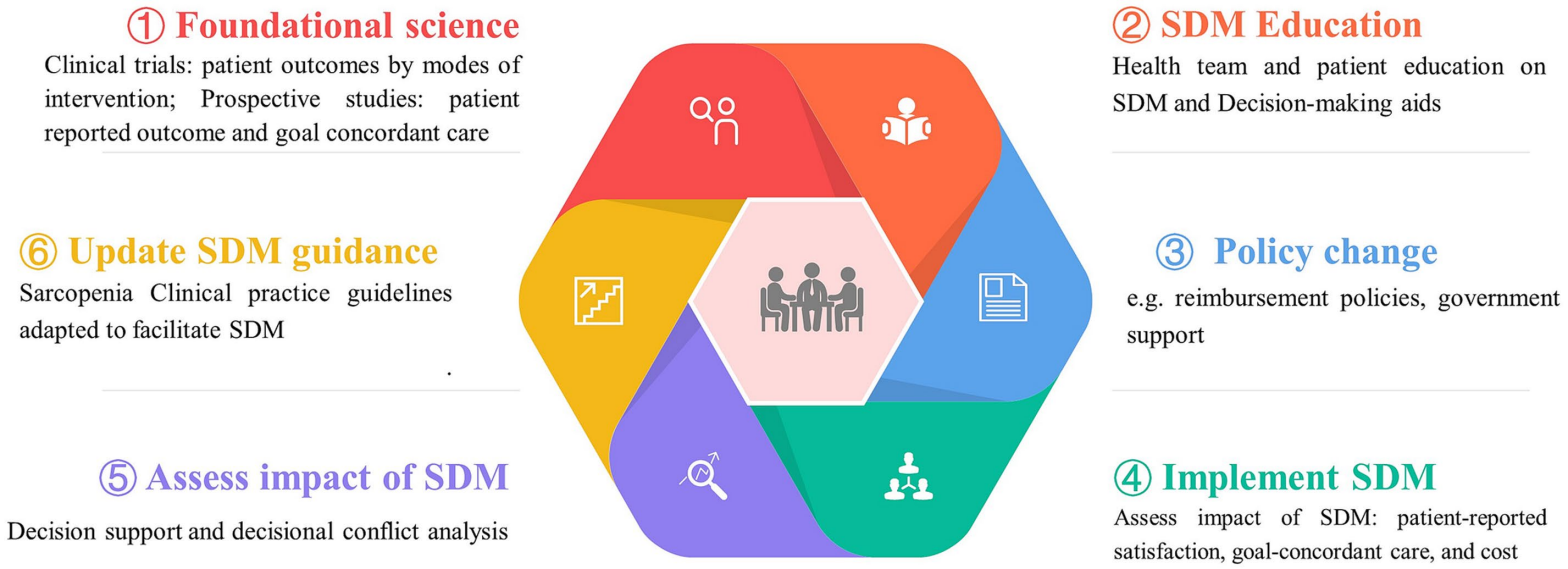
Optimizing the approach to shared decision making in sarcopenia management. Optimizing shared decision making in the treatment of sarcopenia involves addressing a complex array of challenges, including gathering quantitative and qualitative research data, enhancing patient-provider communication, revising current policies, and assessing the impact of SDM on patient-centered outcomes.

Facilitators and barriers to high-quality SDM have been extensively studied and confirmed, including factors such as anxiety, cultural background, trust, and other psychodynamic elements (38). Decision-making becomes more complicated for older individuals with sarcopenia and multiple healthcare needs due to factors such as limited resource availability, decline in decision-making abilities, suitability of treatment options, and an increased likelihood of experiencing depression (2, 38, 39). The decision-making process may require negotiations and communication among various healthcare professionals, patients, and their families. Although evidence suggests that numerous older adults patients and their caregivers aspire to engage in decision-making, initiating discussions and sharing preferences can present challenges (2). Therefore, establishing mechanisms that support and promote SDM in sarcopenia care among healthcare providers, patients, and social caregivers is important. A path to optimize the SDM implementation is shown in Figure 2.

Respect and cultural sensitivity are critical factors in the SDM process (2, 40). Chinese patients are more likely to assume a passive role in the decision-making process than patients from other countries are (40). To serve individuals better, assessments and interventions should be selected after considering cultural factors, including cultural preferences and norms. In China, families play a dominant role in decision making. The family is often an extension of the patient and ensures that the patient processes and understands the information. Additionally, community-based rehabilitation, as an extension of hospital and post-hospital rehabilitation (41), has gained importance with continuous government investment in older adults care service facilities, particularly in community-based workouts and rehabilitation equipment. In pursuit of this goal, healthcare teams should be encouraged to conduct regular visits and maintain close communication with the families of patients who choose home-based rehabilitation services and nutritional interventions.

Improving healthcare professionals’ understanding of SDM concepts is crucial to enhance their applications in clinical practice. This challenging situation demands not only a solid comprehension of the underlying SDM principles, but also familiarity with various methods for treating sarcopenia (42, 43). Excellent communication skills during patient interactions and interdisciplinary settings, along with exceptional social skills, are required to grasp individual needs and discuss available options without bias (44). Furthermore, facilitating discussions in easily understandable languages to prioritize various interventions is of the utmost importance. Training health professionals and patients (at all stages) could strengthen SDM (2).

Establishing collaborative teams is a crucial factor driving the implementation of SDM mechanisms. Effective communication among health care professionals is essential in SDM to provide patients with structured information. One study revealed that time constraints during SDM implementation in clinics pose a significant challenge, leading to limitations in practice (45). Nurses continue to constitute the majority of healthcare professionals (46). Considering the extended assessment time required for sarcopenia, a nurse-led evaluation may be a feasible solution. Moreover, healthcare professionals with SDM knowledge, skills, and positive attitudes play a significant role in implementing and promoting the process (46).

Moreover, decision coaching, clinical counseling, and patient decision aids (PDAs) are rapidly developing (2). PDAs have demonstrated greater effectiveness than that demonstrated by usual care in reducing decisional conflicts, increasing patient participation, and enhancing knowledge of treatment options and potential outcomes (47, 48). Visualization techniques allow PDAs to present personalized and tailored information that is easy to access before the actual physician-patient interaction (49). Evidence-based patient information (EBPI) is a prerequisite for informed decisions. PDAs and EBPI are commonly used in surgical, oncological, and screening decision making. Guidelines for developing PDAs have been published, including criteria for EBPI and International Patient Decision Aid Standards (50, 51). Unfortunately, PDAs that fully comply with the EBPI criteria in the context of sarcopenia management. Additionally, non-English versions of SDM tools validated through standard cross-cultural validation approaches are lacking (52).

## Conclusion

Shared decision making is crucial but underexplored in sarcopenia management. Simply educating patients about exercise falls short. Developing SDM tools is vital to understand patient preferences, needs, and values. Implementing thoughtful SDM and exercise plans shifts the decision-making model in sarcopenia treatment from paternalism to patient-centered care and partnership of care.

## Author contributions

KA: Software, Supervision, Validation, Writing – original draft. ZW: Visualization, Writing – original draft. YQ: Data curation, Visualization, Writing – original draft. MP: Data curation, Validation, Writing – original draft. LZ: Conceptualization, Project administration, Writing – review & editing. ZA: Methodology, Resources, Writing – review & editing. SL: Funding acquisition, Methodology, Supervision, Validation, Writing – review & editing.
